# Theoretical Foundations to the Impact of Dog-Related Activities on Human Hedonic Well-Being, Life Satisfaction and Eudaimonic Well-Being

**DOI:** 10.3390/ijerph182312382

**Published:** 2021-11-25

**Authors:** Ana Maria Barcelos, Niko Kargas, John Maltby, Sophie Hall, Phil Assheton, Daniel S. Mills

**Affiliations:** 1 School of Life Sciences, University of Lincoln, Lincoln LN6 7TS, UK; dmills@lincoln.ac.uk; 2 School of Psychology, University of Lincoln, Lincoln LN6 7TS, UK; nkargas@lincoln.ac.uk; 3 Department of Neuroscience, Psychology and Behaviour, University of Leicester, Leicester LE1 7RH, UK; jm148@leicester.ac.uk; 4 School of Medicine, University of Nottingham, Nottingham NG7 2RD, UK; sophie.hall@nottingham.ac.uk; 5 Department of Statistics, StatsAdvice.com, Ltd., 10551 Berlin, Germany; phil@statsadvice.com

**Keywords:** dog ownership, dog-related activities, eudaimonic, hedonic, human-animal interactions, life satisfaction, mental health, well-being

## Abstract

Cross-sectional comparisons of well-being between dog owners and non-owners commonly generate inconsistent results. Focusing on the uniqueness of the relationship might help address this issue and provide a stronger foundation for dog-related psychotherapeutic interventions. This study aims to evaluate the impact of dog-related activities (e.g., exercising the dog) on owner hedonic well-being, life satisfaction and eudaimonic well-being. It was also hypothesised that psychological closeness to the dog would affect these well-being outcomes. For this study, 1030 dog owners aged over 18 years old answered an online questionnaire about the impact of 15 groups of dog-related activities on their well-being. Ordinal regressions were used to estimate the mean response (and its uncertainty) for each outcome, while conditioning for psychological closeness to the dog and controlling for several key covariates. Tactile interactions and dog playing were significantly more beneficial than other activities for hedonic well-being, and dog training and dog presence for eudaimonic well-being. In contrast, dog health issues and behavioural problems were linked to decrements in these well-being outcomes. Higher psychological closeness to the dog predicted greater improvement in well-being in positive dog-related activities. Our quantitative study validates the general findings of previous qualitative work and lays the groundwork for future longitudinal studies.

## 1. Introduction

Despite frequent reports of the benefits of pet ownership to human well-being based on qualitative studies, quantitative findings based largely on cross-sectional comparisons between owners and non-owners are inconsistent [1,2,3,4,5]. These mixed results highlight not only the complexity of the pet-human relationship but also the need for theoretical frameworks that account for the diversity of the relationship [4]. For example, although McNicholas et al. [6] have proposed a range of mechanisms whereby pet ownership might have a direct, indirect (via enhancement of social interactions) or no effect at all on human well-being, this is clearly not a comprehensive list. By contrast, Gee et al. [7] have suggested that a biopsychological model may explain the benefits of dog-human interaction. However, while this might seem more comprehensive, it lacks detail concerning the specific behaviours associated with ownership that impact human well-being. For example, are the benefits related to dog walking, tactile interactions, the routine of looking after the animal, or simply to the animal’s presence? Similar questions are being increasingly asked, not only in studies of pet ownership [8,9,10,11,12,13], but also in experiments featuring human-animal interaction [14,15,16,17]. By addressing the specific activities/situations associated with pet ownership, we can begin to objectively address the psychological basis to any benefits.

Barcelos et al. [18] have recently proposed a framework that describes 58 dog-related activities/situations (e.g., dog walking, feeding the dog and dog aggression), organised into 15 themes (e.g., exercise, providing for the dog and unwanted behaviours), and their impact on hedonic well-being (positive and negative affect) [19,20], life satisfaction [21] and eudaimonic well-being (i.e., six areas of life functioning: autonomy, purpose in life, personal growth, control over own environment, self-acceptance and positive relations with others) [22,23] of UK dog owners. This thematic framework has also been shown to be relatively robust in other cultures (e.g., Brazilian dog owners) [24], amongst a more neurodiverse population (e.g., autistic adults dog owners) [25], and even in other species (e.g., cat owners) [26], illustrating how pet ownership can be deconstructed into a range of related activities that might positively or negatively impact human well-being. Besides accommodating the uniqueness of the pet-owner relationship, this approach, unlike many others, considers both hedonia and eudaimonia, as recommended for investigations of well-being, given their complementary psychological functions. Hedonic pursuit is associated more with immediate well-being (e.g., everyday affect), while eudaimonic is more closely associated with longer-term well-being (e.g., life engagement) [27]. Findings of other studies also indicate that the psychological closeness between owner and dog could be an important moderator of owner well-being outcomes [28,29]. The latter found a positive correlation with well-being (i.e., life satisfaction and happiness) and a negative correlation with psychological distress, and in the study by Cavanaugh et al. [28], owners who reported higher psychological closeness to their dog tended to be more satisfied with their lives.

Having used qualitative methods to build a framework of dog owner interactions which impact on their well-being [18], the aim of the current study was to use quantitative methods to deduce the significance of the reported relationships between types of dog owner activity and their impact on owner well-being in a wider population [30]. Furthermore, given the potential importance of psychological closeness on the proposed effects, we included a measure of this in order to illustrate the importance of psychological closeness in relation to different types of activity.

## 2. Materials and Methods

### 2.1. Ethical Approval

The study was approved by the Ethics Committee of the University of Lincoln (protocol code 2021_0503). Electronic informed consent was obtained from participants prior to their participation.

### 2.2. Questionnaire Design and Structure

Participants completed an online questionnaire (QualtricsTM) divided into two sections: (1) general questions about the participant and their dog, used as covariates in the statistical analysis, and (2) questions about the impact of 15 groups of dog-related activities on participants’ hedonic well-being, life satisfaction and eudaimonic well-being. A total of eleven aspects of well-being were self-reported in relation to these activities: life satisfaction [21], four elements of hedonic well-being [31,32,33] and six elements of eudaimonic well-being [22].

Most questions of the survey were not mobile friendly. Therefore, participants were advised at the introduction of the survey to answer the survey from a desktop and not from their phones. The questionnaire is available in the Supplementary Material.

### 2.3. Measurements

#### 2.3.1. Dog-Related Activities/Situations

The dog-related activities (Table 1) were based on our previous framework [18,24,25]. To decrease potential order effects, the order of the questions about dog-related activities (for example, how does ‘dog playing’ impact on your satisfaction with your own life?) was randomised for each participant. The impact of each of these activities on the 11 well-being outcomes was assessed using a 7-point Likert response (−3 worsen a lot, −2 worsen moderately, −1 worsen a bit, 0 no impact, +1 improve a bit, +2 improve moderately, +3 improve a lot).

#### 2.3.2. Life Satisfaction and Hedonic Well-Being

Life satisfaction was referred to as ‘satisfaction with your own life’. The four aspects of hedonic well-being were described in terms of states: (1) ‘positive affect of high arousal (e.g., happiness, excitement, joy, fun, activation, etc)’, (2) ‘positive affect of low arousal (e.g., calmness, relaxation, serenity, peacefulness, etc)’, (3) ‘negative affect of high arousal (e.g., stress, annoyance, worry, frustration, anxiety, anger, etc)’, and (4) ‘negative affect of low arousal (e.g., sadness, tiredness, unhappiness, guilt, feeling low, grief, etc)’.

#### 2.3.3. Eudaimonic Well-Being

The six elements of eudaimonia considered were: (1) ‘autonomy (your independence, freedom from others’ approval)’, (2) ‘your control over situations/events in your life or in your surroundings’, (3) ‘personal growth (your growth or achievement of your potentials)’, (4) ‘positive relations with others (good social relations with other people)’, (5) ‘purpose in life (having aims/goals in life)’, and (6) ‘self-acceptance (accepting yourself, the good and bad in you)’.

#### 2.3.4. Closeness to the Dog

Finally, the level of psychological closeness to the dog was measured with an adapted version of the Inclusion of Other in the Self scale [34], a visual scale, ranging from 1 to 7, originally developed to assess interpersonal closeness.

### 2.4. Participants

A power analysis was conducted prior to data collection. Aiming for 99% chance of reaching narrow confidence intervals equivalent to ≤20% of the one-point distance between the options given to participants (e.g., improve moderately and improve a lot) in each activity, a sample of 1000 dog owners was estimated. The power analysis was based on the estimated marginal means of an ordinal regression analysis applied to each of the 1000 simulations. Each simulation assumed weak correlations (R = 0.1) between all pairs of covariates and moderate correlations (R = 0.3) between the DV and three of the covariates (zero correlation between DV and the remaining covariates). The model was further challenged in the power analysis by capturing an unequal spread of responses across the seven ordinal response categories (the seven categories were chosen to have marginal probabilities of selection of 0.01, 0.01, 0.01, 0.07, 0.1, 0.2 and 0.6, respectively).

Using convenience and snowball sampling, volunteer participants were recruited via social media (i.e., Facebook, Twitter, Quora, Reddit, WhatsApp) and local media (i.e., University of Lincoln News). To be included, they needed to be at least 18 years old and own a dog.

### 2.5. Statistical Analysis

Estimated means were calculated using ordinal probit regression models on the R packages ‘ordinal’ (v.2019.12-10) and ‘emmeans’ (v.1.6.1). The following participants’ characteristics were included as covariates in the statistical models, as they could influence owners’ well-being: age (continuous variable [35]), gender (female, male, non-binary [36]), country (UK, US, other [37]), living alone (yes/no [38]), on the autism spectrum (yes/no [39]), diagnosed or experiencing a mental health problem (yes/no [40]), and level of psychological closeness to the dog (1 to 7 [29]). Dogs’ characteristics were also controlled in the models, as they might influence the performance of dog-related activities and/or human well-being (e.g., dog size can affect dog walking performance [41]): sex of the dog(s) (female, male, mixed—e.g., one female and one male in the household was coded as mixed), young dog—under one year old (yes/no), senior dog—10 years old or more (yes/no), reproductive status (intact, neutered/spayed, mixed—i.e., having both intact and neutered dogs in the household was coded as mixed), very small/small dog (yes/no), and very large/giant dog (yes/no).

**Figure 1:**
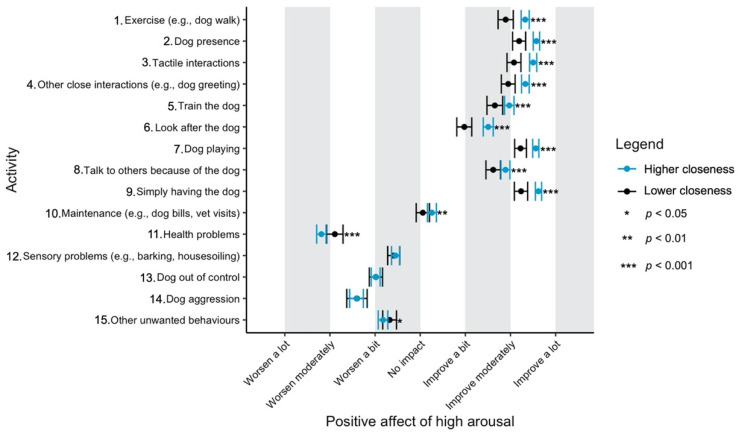
Reported impact of dog-related activities on positive affect of high arousal, at lower and higher level of closeness to the dog.

**Figure 2:**
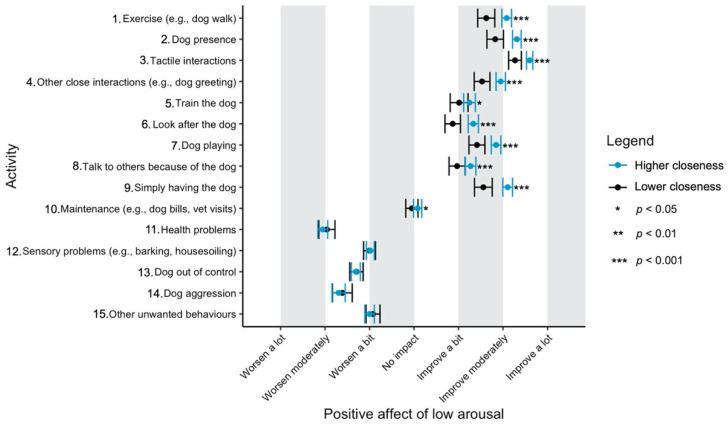
Reported impact of dog-related activities on positive affect of low arousal, at lower and higher level of closeness to the dog.

**Figure 3:**
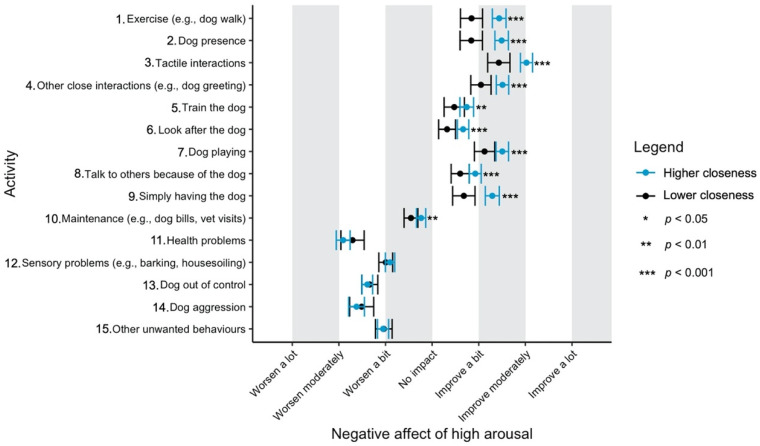
Reported impact of dog-related activities on negative affect of high arousal, at lower and higher level of closeness to the dog.

**Figure 4:**
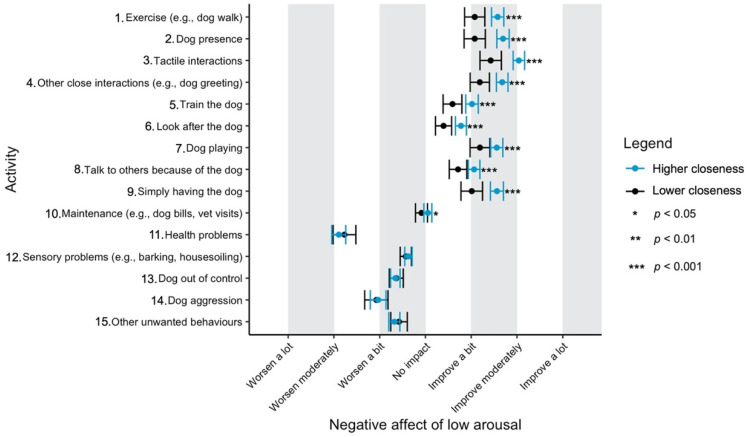
Reported impact of dog-related activities on negative affect of low arousal, at lower and higher level of closeness to the dog.

**Figure 5:**
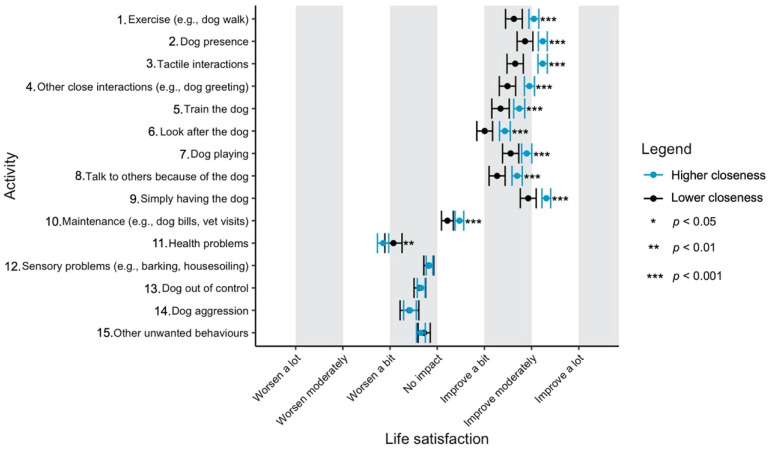
Reported impact of dog-related activities on life satisfaction, at lower and higher level of closeness to the dog.

**Figure 6:**
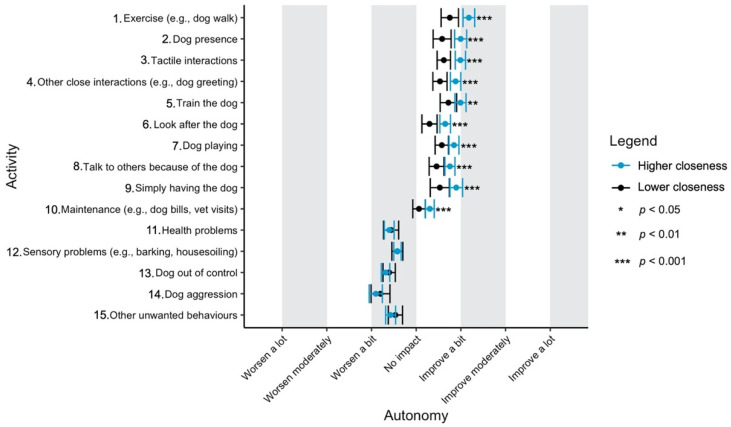
Reported impact of dog-related activities on autonomy, at lower and higher level of closeness to the dog.

**Figure 7:**
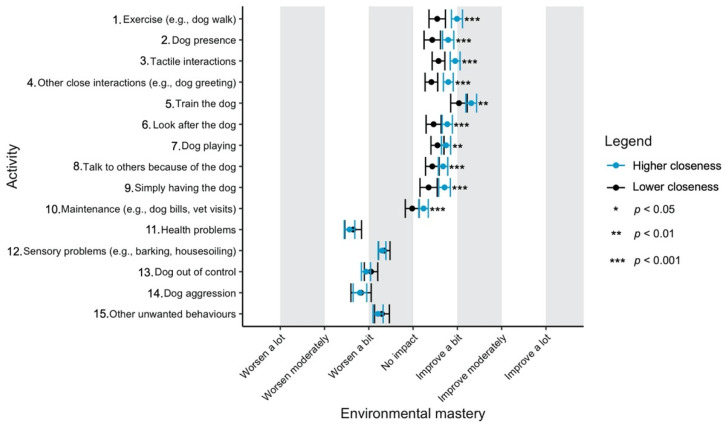
Reported impact of dog-related activities on environmental mastery, at lower and higher level of closeness to the dog.

**Figure 8:**
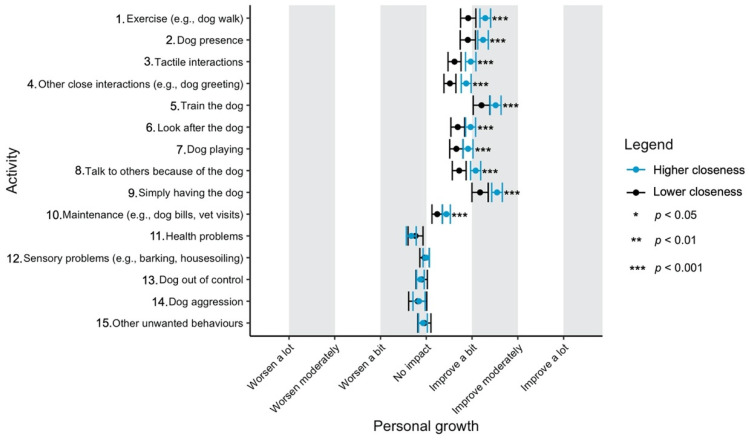
Reported impact of dog-related activities on personal growth, at lower and higher level of closeness to the dog.

**Figure 9:**
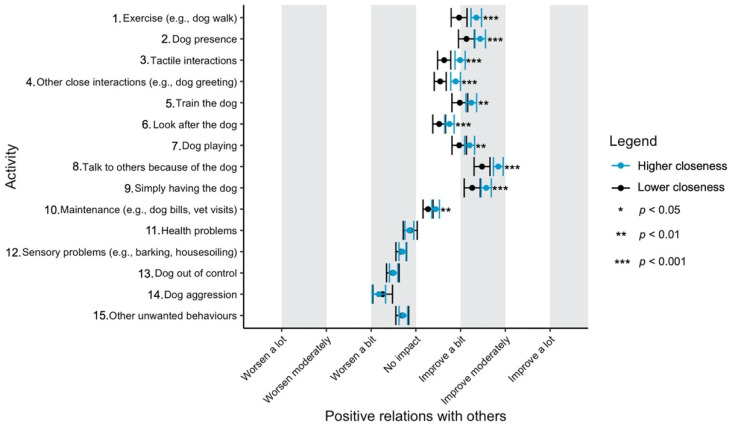
Reported impact of dog-related activities on positive relations with others, at lower and higher level of closeness to the dog.

**Figure 10:**
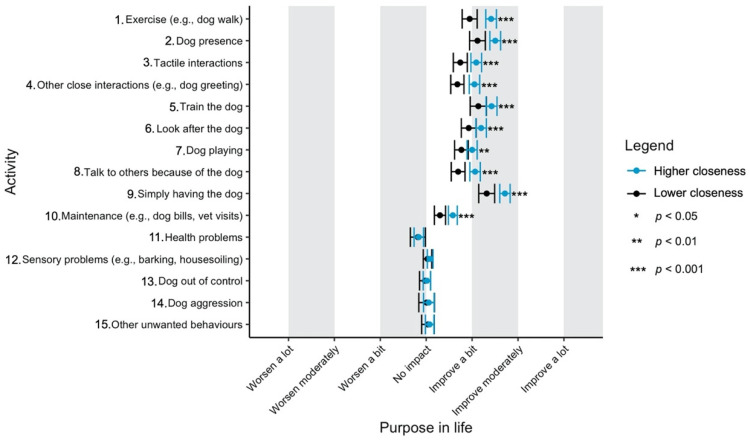
Reported impact of dog-related activities on purpose in life, at lower and higher level of closeness to the dog.

**Figure 11:**
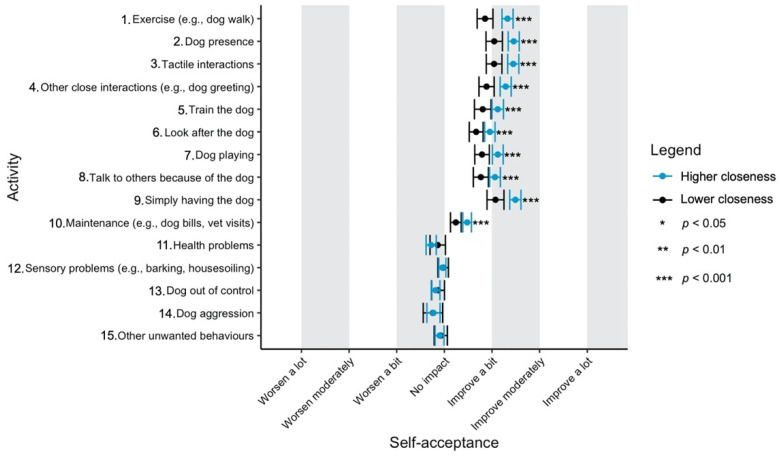
Reported impact of dog-related activities on self-acceptance, at lower and higher level of closeness to the dog.

Given the focus of this study on how dog-related activities differ in their impact on well-being, we have used a graphical approach to present our results (Figures 1–11—presented later in the manuscript). We show the estimated means for each pair of activity and well-being outcome (e.g., life satisfaction rating due to exercise with the dog versus life satisfaction due to dog playing). Such an approach facilitates the visualisation of statistical differences between dog-related activities. Plots (Figures 1–11) show, through error bars (99% CI), the regressed well-being outcomes of each dog-related activity for both owners with lower (score 2—lower quartile) and higher (score 6—upper quartile) psychological closeness to their dogs. Plots were created using the R package ‘ggplot2’ (v.3.3.3). Due to the large number of ordinal regressions performed, a more conservative confidence interval (i.e., 99%) has been used in the error bars in Figures 1–11 to provide some mitigation of Type I errors.

## 3. Results

### 3.1. Demographics

Of the 2041 people who started the survey, 1030 met the inclusion criteria and completed the entire survey (50.5%). Respondents (Table S1) were aged 18 to 82 years old (mean 39.7 years, sd = 14.1), were mostly female (83.9%), and were mostly living in the US (38.4%) or UK (33.9%). Further details of the participants and their dogs are available in the Supplementary Material (Table S1). Of those respondents who did not complete the questionnaire, 598 had similar demographics to the actual participants of the study: 18–79 years old (mean 38.9, sd = 13.4), mostly female (79.9%), and mostly living in the US (38.3%) or UK (35.6%). Demographic information of the remaining 413 respondents is not available, as those individuals either did not meet the inclusion criteria, being redirected to the end of the survey, or stopped at the introductory stage due to a warning message telling them to answer the survey from a desktop and not from their phones

### 3.2. Overview of the Reported Impact of Dog-Related Activities on Well-Being

Nine dog-related activities tended to be reported as predominantly positive for well-being across the aspects of well-being assessed (Figure 1, Figure 2, Figure 3, Figure 4, Figure 5, Figure 6, Figure 7, Figure 8, Figure 9, Figure 10 and Figure 11): exercise with the dog (e.g., walking); the presence of the dog; tactile interactions with the dog; other close interactions (e.g., dog greeting the owner); training the dog; looking after the dog (e.g., feeding); dog playing; talking to others because of the dog; simply having the dog. These activities will be referred to as ‘positive activities’ from hereon. In contrast, five activities were mainly detrimental to participant well-being: dog health problems (e.g., dog being sick); sensory behaviour problems (e.g., dog barking); lacking control over the dog (e.g., dog pulling on the lead); dog aggressive behaviours; other dog unwanted behaviours. These will be referred to as ‘negative activities’ from hereon. One activity (maintenance of the dog, e.g., taking dog to the vet) mainly had no to little impact across participants’ well-being.

In Figure 1, Figure 2, Figure 3, Figure 4, Figure 5, Figure 6, Figure 7, Figure 8, Figure 9, Figure 10 and Figure 11, the non-overlap of error bars between different dog-related activities indicates that the activities compared are significantly different (*p* < 0.01). For example, in terms of the ‘positive affect of high arousal’ (Figure 1), dog playing increases happiness significantly more than looking after the dog in both owners with higher and lower closeness to their dogs. To avoid repetition of information provided in the figures, only the most relevant activities—highest/lowest score in our sample and overlapping the least with other activities—are reported in the text of each figure. However, this does not imply that this activity is significantly different to all of the others, as it could still overlap with other activities in the figure. The overlap of activities indicates that they might have a similar impact on the respective well-being outcome. Finally, the estimated means for each pair of dog-related activity and well-being outcome is available in the Supplementary Material (Tables S2–S12).

### 3.3. Relationship between Closeness to the Dog and Reported Well-Being Outcomes

On average, participants reported moderate-high level of psychological closeness to their dogs (mean = 4.82, sd = 1.89). The effect of closeness to the dog is illustrated in Figure 1 and Figure 2. Asterisks next to the error bars indicate the level of significance of this variable. Overall, when rating ‘positive activities’ or ‘maintenance of the dog’, owners who were closer to their dogs reported greater improvement in well-being outcomes than owners with lower closeness. By contrast, in relation to ‘negative activities’, the difference in well-being between owners with higher or lower closeness to their dogs were generally not significant. The exceptions to this were in relation to ‘health problems’, being more negatively rated for its impact on ‘positive affect of high arousal’ and ‘life satisfaction’ by owners who were closer to their dogs (Figure 1 and Figure 5); and in relation to ‘other unwanted behaviours’, being more detrimental for ‘positive affect of high arousal’ in owners with higher closeness to their dogs (Figure 1). Full statistical details for the independent variable ‘closeness to the dog’ and the confidence intervals of well-being outcomes at lower and higher closeness to the dog are available in the Supplementary Material (Tables S13–S33).

### 3.4. Effect of Dog-Related Activities on Specific Well-Being Outcomes

#### 3.4.1. Positive Affect of High Arousal (e.g., Happiness, Excitement, Joy)

‘Dog playing’, ‘simply having the dog’ and ‘dog presence’ were the most positive activities for happiness, whereas ‘health problems’ and, secondly, ‘dog aggression’ were the most detrimental ones (Figure 1).

#### 3.4.2. Positive Affect of Low Arousal (e.g., Calmness, Relaxation, Peacefulness)

‘Tactile interactions’ and ‘dog presence’ led the rating scores for calmness improvement. ‘Health problems’ and ‘dog aggression’ were, again, the most detrimental activities (Figure 2).

#### 3.4.3. Negative Affect of High Arousal (e.g., Stress, Anger, Frustration)

‘Tactile interactions’ and, secondly, ‘dog playing’ and ‘other close interactions’ were reported to improve most the negative affect of high arousal (Figure 3). ‘Health problems’ and ‘dog aggression’ were, again, the worst for this aspect of well-being.

#### 3.4.4. Negative Affect of Low Arousal (e.g., Sadness, Feeling Low, Frustration)

Similar to what has been described in terms of the negative affect of high arousal, ‘tactile interactions’, ‘other close interactions’ and ‘dog playing’ were the most positive for negative affect of low arousal (Figure 4). Again, ‘health problems’, followed by ‘dog aggression’, were the worst for this aspect of owners’ well-being.

#### 3.4.5. Life Satisfaction

Similar to positive affect of high arousal, ‘simply having the dog’ and ‘dog presence’ were the activities that improved most the owners’ life satisfaction (Figure 5), whereas ‘health problems’ was the activity that worsened this well-being outcome the most.

#### 3.4.6. Autonomy

All ‘positive activities’ were rated as having a very similar positive impact on owners’ autonomy. However, ‘exercising’ with the dog seems to be slightly better than other activities (Figure 6). The ‘negative activities’ were also rated similarly, but ‘dog aggression’ had the most negative effect, particularly among owners with higher closeness to their dogs.

#### 3.4.7. Environmental Mastery

‘Training the dog’ was the activity reported to most improve an owner’s sense of environmental mastery (Figure 7). In contrast, ‘health problems’ and ‘dog aggression’ were the worst for environmental mastery.

#### 3.4.8. Personal Growth

According to owner ratings, ‘training the dog’ and ‘simply having the dog’ were the most beneficial activities for personal growth (Figure 8). Although the negative activities did not seem to have a considerable impact on personal growth, ‘health problems’ was the most detrimental for this well-being outcome, particularly for owners with higher closeness to their dogs.

#### 3.4.9. Positive Relations with Others

Positive relations with others was reported to improve most due to ‘talk to others because of the dog’, followed by ‘simply having the dog’, ‘dog presence’, ‘dog training’, ‘dog playing’ and ‘exercise’ (Figure 9). As with autonomy, ‘dog aggression’ was the most detrimental event impacting on this aspect of well-being.

#### 3.4.10. Purpose in Life

‘Simply having the dog’, ‘training the dog’ and ‘dog presence’ were reported to increase owners’ purpose in life the most. Such as observed in personal growth, ‘health problems’ was the only activity significantly bad for purpose in life in both closer and more distant owners (Figure 10).

#### 3.4.11. Self-Acceptance

As with autonomy, the ratings of the ‘positive activities’ were very similar to each other. Still, ‘dog presence’, ‘tactile interactions’ and ‘simply having the dog’ seem to be the most beneficial for self-acceptance. ‘Dog aggression’ was the only activity significantly detrimental for self-acceptance independently of level of closeness to the dog (Figure 11), although high closeness made ‘health problems’ more likely to be associated with a negative impact.

## 4. Discussion

The findings indicate that different dog-related activities have varying impacts on human hedonic well-being, life satisfaction and eudaimonic well-being, as suggested in our previous qualitative work [18,24,25]. These specific findings have the potential to lay the foundation for more targeted psychotherapeutic interventions using owners’ own pet dogs and, possibly, also assistance animals in AAI (e.g., positive tactile interaction with a dog to help decrease negative affect of high arousal in a patient). We suggest that specific aspects of human well-being might be more effectively boosted using a more tailored activity-based approach, based on the leading activities in Figure 1, Figure 2, Figure 3, Figure 4, Figure 5, Figure 6, Figure 7, Figure 8, Figure 9, Figure 10 and Figure 11. Further these results provide important insight into why the reported benefits of pet ownership are so inconsistent [1,2,3,4,5]; i.e., different owners will engage in different activities and thus receive varying amounts of benefit or cost to diverse types of well-being. The activities’ high variability in their level of impact across different well-being outcomes also highlights the importance of a comprehensive assessment of well-being in this type of work [27]. In this way, the potential benefits or costs associated with a specific activity are less likely to be missed. Another important finding was the potential moderating role of the closeness of the relationship between the owner and dog, on many of the potential effects.

### 4.1. Closeness to the Dog

A higher level of closeness to the dog was associated with greater perceived benefits from all ‘positive activities’ and ‘maintenance of the dog’ in all aspects of well-being, which might explain why owners who are closer to their dogs have greater well-being [28,29] and are more satisfied with their relationship with their dogs than other owners [42]. In contrast, ‘negative activities’ were generally scored similarly by owners regardless of their closeness. In other words, dog behavioural and health problems generally worsen the well-being of a dog owner to a similar degree. These findings have important implications for the management of people’s mental health: since closer owners seem to benefit more from ‘positive activities’, increased frequency/duration of those activities (e.g., walking the dog more often) are likely to be much more efficacious for them. It is also worth speculating that when these activities are used within an AAI context, their impact may be less than for an owner, until a close bond is formed. Thus, focusing on building emotional closeness between a client and therapeutic animal may be important to the efficacy of a proposed activity. By contrast, psychological interventions focused on decreasing ‘negative activities’ (e.g., dog behaving less aggressively) appear to have a similar effect on the owner regardless of their emotional closeness.

### 4.2. Hedonic Well-Being and Life Satisfaction

Positive interactions with the dog (e.g., touching, being greeted by the animal) and routine-like activities (e.g., walking, feeding) were rated as beneficial to owners’ hedonic well-being, which is consistent with the findings of Barcelos et al. [18,25] and Corrêa et al. [24]. Tactile interactions, in particular, led the rating score in three aspects of hedonic well-being, increasing owners’ calmness and decreasing both stress and sadness. Indeed, it has been suggested that human-dog tactile interactions are a major component of the ‘pet effect’ [15], being associated with lower human cortisol [43,44,45], blood pressure, heart rate [15], and higher oxytocin [43,44]. Playing with the dog or watching the animal play were also very important to three aspects of hedonic well-being, improving owners’ happiness, stress and sadness. Horowitz and Hecht [46] reported that, from their observations, 61% of dog-human playful interactions appear to elevate owners’ positive affect. Human cortisol has also been reported to decrease after ‘low-key playful interactions’ with dogs [44] and qualitative studies have consistently reported the hedonic benefits of dog play (e.g., is enjoyable/fun, improves sadness, stress [18,24,25,47,48]. However, in a cross-sectional study, owners’ reported frequency of dog playing was not correlated with well-being scores (e.g., stress, psychosomatic symptoms) [49], but this may reflect the issue of less precise measurement of well-being in many studies, that we commented on in the introduction to this discussion.

Life satisfaction was rarely mentioned by participants in our previous qualitative studies [18,24,25], however all ‘positive activities’ were rated here as positive to life satisfaction. Indeed, simply having a dog seems to improve life satisfaction [50], even if this is not something that often comes to mind in interviews or focus groups. In the current study, the mere ‘presence of the dog’ led the rating scores for life satisfaction and positive affect of high arousal. The benefits of the dog’s presence for humans is one area of human-animal interaction where the scientific results are fairly consistent: being associated not only with beneficial neurophysiological changes [16,51] but also with improvements in self-reported well-being [8,11,14,18,24,25]. By contrast, dog health problems (e.g., disease, injury) and behavioural problems, particularly dog aggression, were reported to worsen owners’ hedonic well-being and life satisfaction. Caring for sick animals is a burden and source of stress for pet owners [52,53,54,55,56]; likewise, pet behaviour problems undermine owner well-being [48,57,58] and increase dissatisfaction with their animal [42]. Thus, interventional strategies that minimise detrimental activities and augment positive ones will likely improve owners’ hedonic well-being and life satisfaction.

### 4.3. Eudaimonic Well-Being

Exercising the dog (e.g., walking), looking after the animal (e.g., feeding) and the presence of the dog were positive across all elements of eudaimonic well-being. Dog walking has been reported to give purpose in life and facilitate positive social relationships [9,59,60]. Caring for an animal can give owners routine, life structure and purpose [59,61,62], even potentially helping in suicide prevention [25,63]. Dog presence/company is an important social lubricant [60,64,65,66] and is potentially a source of social support [45]. Interestingly, dog training led the rating score of most eudaimonic elements (e.g., environmental mastery, personal growth). Positive effects from dog training on eudaimonia have been reported in qualitative studies of owners (e.g., [18,24,25]), and in relation to formal dog training programs (e.g., with prisoners, or soldiers with post-traumatic stress disorder). This might be because improving participants’ skills, autonomy, trust in others and emotional control might give them a sense of achievement and self-development [67,68,69,70,71].

Interestingly, while positive activities were universally beneficial, negative events (e.g., health problems, dog aggression) had little to no impact on personal growth, purpose in life and self-acceptance; the significant detrimental effects were mostly related to autonomy, environmental mastery and positive relations with others. The latter negative results might therefore be linked largely to the stress and burden of these activities in the lives of pet owners in terms of owners being unable to make independent choices (autonomy), manage everyday affairs (environmental mastery), and due to interpersonal conflicts (relation with others), e.g., frequent disputes with other people because of dog showing aggression [48,52,53,54,55,56,57,58].

### 4.4. Strengths and Limitations

The strengths of this study include its large sample size and the comprehensiveness of the dog-related activities and well-being outcomes evaluated, providing rich findings that can be empirically tested in future studies. This study had a very solid foundation, building on the consistency of our previous qualitative investigations [18,24,25]. Two main limitations of the study are its cross-sectional methodology, which does not permit inferences of causality, and the large proportion of female participants (83.9%), a common issue in studies of human-animal bond (see [72]), such as those about dog ownership [73]. Another limitation of the study is the lack of assessment of additional covariates that could have impacted how owners perceive the interactions with their dogs, such as participants’ socioeconomic status [74] and personality traits [75]. Utz [76], for example, found that socioeconomic status is an important confounder in the association between pet ownership and human physical health. Despite these limitations, our study controls for several important covariates (e.g., age, gender, country, age of the dog, size of the dog, etc.) which is not always the case in studies on pet ownership [77].

Future investigations could test the impact of fluctuations in dog-related activities on the well-being of dog owners longitudinally, as a way to assess causality. Additionally, the same methodology could be extended to other pets and non-pet animals, such as those studies which have already been initiated with cats [26]. To increase male participation, targeted recruitment strategies can be applied, such as using more male dog owner pictures in advertisement materials, asking participants to invite male friends to the study, targeting social media groups/venues more populated by men.

## 5. Conclusions

By employing a wider spectrum of hedonic and eudaimonic well-being herein, we may have begun to specify where the greatest impact of various human-dog interactions lies. Overall, different dog-related activities were found to have specific effects on owner well-being. Accordingly, we caution against over-simplified generalisations of a ‘pet effect’ or recommendations of pet acquisition as a psychological aid. A more individualised approach, based on increments or decrements in dog-related activities aiming to improve specific aspects of one’s well-being is likely to result in more predictable outcomes and thus be more fruitful. Attention should also be given to the level of closeness between the person and the dog, as this may have significant moderating effects. Future psychotherapeutic interventions with pet dogs or studies about dog ownership and human well-being could use the findings presented here to provide guidance for their hypotheses and therapeutic goals. We hope that greater understanding of the complexity of the dog-human relationship and the impact that dogs have on human mental health will not only help shape a better human-dog relationship, but also a better society for all.

## Figures and Tables

**Table 1:** Dog-related activities/situations assessed in the questionnaire.

1. **Exercise** (e.g., walking, running, hiking with the dog, etc)	6. **Look after the dog** (e.g., feeding, giving water)	11. **Health problems** (dog health issues, e.g., injury, sickness)
2. **Dog presence** (i.e., the presence/company of the dog)	7. **Dog playing** (i.e., play with the dog or watch the dog play)	12. **Sensory problems** (i.e., behavioural issues that disturb owner’s senses, e.g., dog barking, house soiling)
3. **Tactile interactions** (i.e., touching or being touched by the dog)	8. **Talk to others because of the dog** (e.g., say hello to others because of dog)	13. **Dog out of control** (i.e., loss or lack of control over the dog, such as when the dog pulls on the lead, does not respond to recall)
4. **Other close interactions** (e.g., dog greeting, talking to the dog)	9. **Simply having the dog** (i.e., being a dog owner)	14. **Dog aggression** (e.g., growling, trying to bite, biting)
5. **Train the dog** (e.g., commands, housetraining)	10. **Maintenance** (i.e., involvement in indirect tasks to provide for the dog, such bills, vet visits, purchases)	15. **Other unwanted behaviours** (e.g., destruction of items, attention seeking, separation-related problems, etc)

## Data Availability

The data that support the findings of this study are openly available in Open Science Framework at https://osf.io/n6m2k/?view_only=3e70af2059564120a0383f1ed8937b30, accessed on 20 November 2021.

## Supplementary Materials

The following are available online at https://www.mdpi.com/article/10.3390/ijerph182312382/s1, Questionnaire, Table S1: Characteristics of participants (*n* = 1030) of the study and of their dogs, Tables S2–S12: Estimated means and sd for each pair of activity and well-being outcome, Tables S13–S23: Statistics of the independent variable ‘closeness to the dog’ in the ordinal regressions, Tables S24–S33: Confidence intervals of each activity at lower and higher ‘closeness to the dog’.
