# Supplementary material for: Theoretical Foundations to the Impact of Dog-Related Activities on Human Hedonic Well-Being, Life Satisfaction and Eudaimonic Well-Being
Source: Int J Environ Res Public Health. 2021 Nov 25;18(23):12382. doi: 10.3390/ijerph182312382 (PMC8656591; doi:10.3390/ijerph182312382)
Supplement: Supplementary file 1 [file ijerph-18-12382-s001.zip › ijerph-1453535-supplementary.pdf]

Table S1. Characteristics of participants (n=1030) of the study and of their dogs

Category	n	% or mean(sd)	Category	n	% or mean (sd)
Gender			Mental health problem		
Female	864	83.9%	*Yes (and diagnosed)*	368	35.7%
Male	139	13.5%	*Yes (not diagnosed)*	110	10.7%
Non-binary	27	2.6%	Mental health problem		
Age			*Anxiety*	357	34.7%
18-82 years old	1030	39.7(14.1)	*Depression*	334	32.4%
			Other (e.g., PTSD schizophrenia)	121	11.7%
18-29	302	29.3%			
30-39	294	28.5%	Closeness to dog		
40-49	169	16.4%	*1-7*	1030	4.8(1.9)
50-59	140	13.6%	Number of dogs		
60-69	94	9.1%	*One*	630	61.2%
70 or older	31	3.0%	*Two or more*	400	38.8%
Country			Sex of the dog(s)*		
United States	396	38.4%	*Female*	603	58.5%
United Kingdom	349	33.9%	*Male*	655	63.6%
Other	285	27.7%	Age of the dog(s)*		
Canada	65		*Young (1-11 months)*	155	15.0%
Brazil	26		*Adult (1-9 years)*	816	79.2%
Germany	19		*Senior (10 or more)*	269	26.1%
Australia	19		Reproductive status*		
France	10		*Neutered*	833	80.9%
Others (< 10 each)	146		*Intact*	306	29.7%
Living alone			Size of the dog*		
Yes	191	18.5%	*Very small/small*	375	36.4%
Autism spectrum disorder			*Medium/large*	594	57.7%
Yes	47	4.6%	*Very large/giant*	228	22.1%

* Participants could select more than one choice for this variable. Therefore, the percentage sum is above 100%.

Descriptive statistics:

Note: estimated means, sd and median for each pair of activity and well-being outcome

Table S2. Estimated means and sd for positive affect of high arousal in the different activities

Activity	Mean	SD
Simply having the dog	2.496	0.840
Maintenance (e.g., dog bills, vet visits)	0.208	1.017
Health problems	-2.102	1.029
Dog presence	2.455	0.835
Exercise (e.g., dog walk)	2.185	1.028
Tactile interactions	2.377	0.902
Other close interactions (e.g., dog greeting)	2.216	0.950
Train the dog	1.859	1.132
Look after the dog	1.367	1.159
Dog playing	2.455	0.774
Talk to others because of the dog	1.800	1.060
Sensory problems (e.g., barking, housesoiling)	-0.542	0.896
Dog out of control	-0.979	0.948
Dog aggression	-1.403	1.101
Other unwanted behaviours	-0.777	0.857

Table S3. Estimated means and sd for positive affect of low arousal in the different activities

Activity	Mean	SD
Simply having the dog	1.918	1.248
Maintenance (e.g., dog bills, vet visits)	0.058	0.957
Health problems	-2.011	1.075
Dog presence	2.150	1.132
Exercise (e.g., dog walk)	1.933	1.210
Tactile interactions	2.493	0.829
Other close interactions (e.g., dog greeting)	1.813	1.152
Train the dog	1.159	1.355
Look after the dog	1.202	1.209
Dog playing	1.706	1.172
Talk to others because of the dog	1.177	1.230
Sensory problems (e.g., barking, housesoiling)	-0.972	0.902
Dog out of control	-1.294	0.968
Dog aggression	-1.660	1.082
Other unwanted behaviours	-0.977	0.872

Table S4. Estimated means and sd for negative affect of high arousal in the different activities

Activity	Mean	SD
Simply having the dog	1.085	1.612
Maintenance (e.g., dog bills, vet visits)	-0.286	1.042
Health problems	-1.826	1.468
Dog presence	1.271	1.579
Exercise (e.g., dog walk)	1.229	1.584

Tactile interactions	1.826	1.474
Other close interactions (e.g., dog greeting)	1.363	1.427
Train the dog	0.646	1.492
Look after the dog	0.582	1.284
Dog playing	1.363	1.446
Talk to others because of the dog	0.819	1.330
Sensory problems (e.g., barking, housesoiling)	-0.912	1.004
Dog out of control	-1.356	1.116
Dog aggression	-1.568	1.259
Other unwanted behaviours	-1.045	0.982

Table S5. Estimated means and sd for negative affect of low arousal in the different activities

**Activity**	**Mean**	**SD**
Simply having the dog	1.374	1.543
Maintenance (e.g., dog bills, vet visits)	0.020	0.917
Health problems	-1.827	1.503
Dog presence	1.490	1.495
Exercise (e.g., dog walk)	1.398	1.455
Tactile interactions	1.840	1.433
Other close interactions (e.g., dog greeting)	1.522	1.376
Train the dog	0.860	1.404
Look after the dog	0.672	1.249
Dog playing	1.426	1.428
Talk to others because of the dog	0.951	1.317
Sensory problems (e.g., barking, housesoiling)	-0.390	0.811
Dog out of control	-0.646	0.989
Dog aggression	-1.040	1.250
Other unwanted behaviours	-0.645	0.993

Table S6. Estimated means and sd for life satisfaction in the different activities

**Activity**	**Mean**	**SD**
Simply having the dog	2.181	1.055
Maintenance (e.g., dog bills, vet visits)	0.397	0.937
Health problems	-1.083	1.143
Dog presence	2.112	1.054
Exercise (e.g., dog walk)	1.904	1.143
Tactile interactions	2.054	1.092
Other close interactions (e.g., dog greeting)	1.804	1.150
Train the dog	1.593	1.221
Look after the dog	1.310	1.186
Dog playing	1.780	1.143
Talk to others because of the dog	1.555	1.142
Sensory problems (e.g., barking, housesoiling)	-0.165	0.724
Dog out of control	-0.347	0.800
Dog aggression	-0.579	0.979
Other unwanted behaviours	-0.323	0.746

Table S7. Estimated means and sd for autonomy in the different activities

Activity	Mean	SD
Simply having the dog	0.768	1.491
Maintenance (e.g., dog bills, vet visits)	0.239	1.008
Health problems	-0.594	1.023
Dog presence	0.847	1.409
Exercise (e.g., dog walk)	1.027	1.361
Tactile interactions	0.875	1.164
Other close interactions (e.g., dog greeting)	0.768	1.185
Train the dog	0.891	1.266
Look after the dog	0.537	1.227
Dog playing	0.757	1.127
Talk to others because of the dog	0.653	1.170
Sensory problems (e.g., barking, housesoiling)	-0.416	0.826
Dog out of control	-0.667	0.914
Dog aggression	-0.870	1.055
Other unwanted behaviours	-0.529	0.905

Table S8. Estimated means and sd for environmental mastery in the different activities

Activity	Mean	SD
Simply having the dog	0.587	1.377
Maintenance (e.g., dog bills, vet visits)	0.169	1.097
Health problems	-1.407	1.138
Dog presence	0.666	1.324
Exercise (e.g., dog walk)	0.836	1.303
Tactile interactions	0.826	1.121
Other close interactions (e.g., dog greeting)	0.674	1.123
Train the dog	1.216	1.242
Look after the dog	0.676	1.203
Dog playing	0.679	1.072
Talk to others because of the dog	0.600	1.100
Sensory problems (e.g., barking, housesoiling)	-0.682	0.878
Dog out of control	-1.027	1.008
Dog aggression	-1.182	1.122
Other unwanted behaviours	-0.757	0.926

Table S9. Estimated means and sd for personal growth in the different activities

Activity	Mean	SD
Simply having the dog	1.407	1.228
Maintenance (e.g., dog bills, vet visits)	0.389	0.885
Health problems	-0.303	1.022
Dog presence	1.118	1.194
Exercise (e.g., dog walk)	1.168	1.194
Tactile interactions	0.865	1.135

Other close interactions (e.g., dog greeting)	0.760	1.080
Train the dog	1.404	1.217
Look after the dog	0.888	1.111
Dog playing	0.829	1.087
Talk to others because of the dog	0.955	1.114
Sensory problems (e.g., barking, housesoiling)	0.005	0.718
Dog out of control	-0.112	0.806
Dog aggression	-0.168	0.979
Other unwanted behaviours	-0.050	0.800

Table S10. Estimated means and sd for positive relations in the different activities

**Activity**	**Mean**	**SD**
Simply having the dog	1.470	1.209
Maintenance (e.g., dog bills, vet visits)	0.401	0.863
Health problems	-0.139	0.946
Dog presence	1.336	1.230
Exercise (e.g., dog walk)	1.234	1.225
Tactile interactions	0.878	1.146
Other close interactions (e.g., dog greeting)	0.783	1.087
Train the dog	1.151	1.210
Look after the dog	0.689	1.075
Dog playing	1.136	1.139
Talk to others because of the dog	1.716	1.168
Sensory problems (e.g., barking, housesoiling)	-0.294	0.786
Dog out of control	-0.489	0.908
Dog aggression	-0.795	1.075
Other unwanted behaviours	-0.273	0.788

Table S11. Estimated means and sd for purpose in life in the different activities

**Activity**	**Mean**	**SD**
Simply having the dog	1.563	1.203
Maintenance (e.g., dog bills, vet visits)	0.502	0.967
Health problems	-0.172	0.995
Dog presence	1.371	1.218
Exercise (e.g., dog walk)	1.258	1.193
Tactile interactions	0.979	1.170
Other close interactions (e.g., dog greeting)	0.930	1.139
Train the dog	1.304	1.225
Look after the dog	1.122	1.162
Dog playing	0.920	1.111
Talk to others because of the dog	0.938	1.138
Sensory problems (e.g., barking, housesoiling)	0.081	0.653
Dog out of control	0.023	0.798
Dog aggression	0.048	0.874
Other unwanted behaviours	0.077	0.780

Table S12. Estimated means and sd for self-acceptance in the different activities

Activity	Mean	SD
Simply having the dog	1.356	1.232
Maintenance (e.g., dog bills, vet visits)	0.414	0.907
Health problems	-0.242	1.022
Dog presence	1.321	1.216
Exercise (e.g., dog walk)	1.174	1.198
Tactile interactions	1.321	1.204
Other close interactions (e.g., dog greeting)	1.154	1.193
Train the dog	1.010	1.220
Look after the dog	0.868	1.108
Dog playing	1.016	1.155
Talk to others because of the dog	0.968	1.140
Sensory problems (e.g., barking, housesoiling)	-0.027	0.767
Dog out of control	-0.163	0.916
Dog aggression	-0.235	1.014
Other unwanted behaviours	-0.082	0.793

Statistic of the independent variable 'Closeness to the dog' in the ordinal regressions

Note: each line of activity contains the statistic for 'Closeness to the dog' in an ordinal regression

Table S13. Statistics for the variable 'closeness to the dog' when assessing positive affect of high arousal

Activity	B	SE	Z value	Pr(>|Z|)
Simply having the dog	0.139	0.021	6.752	0.000
Maintenance (e.g., dog bills, vet visits)	0.054	0.018	3.000	0.003
Health problems	-0.081	0.021	-3.836	0.000
Dog presence	0.134	0.020	6.659	0.000
Exercise (e.g., dog walk)	0.118	0.019	6.220	0.000
Tactile interactions	0.135	0.020	6.739	0.000
Other close interactions (e.g., dog greeting)	0.111	0.019	5.882	0.000
Train the dog	0.079	0.019	4.203	0.000
Look after the dog	0.124	0.018	6.901	0.000
Dog playing	0.126	0.020	6.273	0.000
Talk to others because of the dog	0.069	0.018	3.814	0.000
Sensory problems (e.g., barking, housesoiling)	0.013	0.019	0.667	0.505
Dog out of control	-0.001	0.020	-0.075	0.940
Dog aggression	-0.003	0.026	-0.099	0.921
Other unwanted behaviours	-0.047	0.023	-2.053	0.040

Table S14. Statistics for the variable 'closeness to the dog' when assessing positive affect of low arousal

Activity	B	SE	Z value	Pr(>|Z|)
Simply having the dog	0.121	0.018	6.570	0.000
Maintenance (e.g., dog bills, vet visits)	0.038	0.018	2.077	0.038
Health problems	-0.025	0.021	-1.174	0.241
Dog presence	0.121	0.019	6.402	0.000
Exercise (e.g., dog walk)	0.104	0.018	5.624	0.000
Tactile interactions	0.118	0.021	5.761	0.000
Other close interactions (e.g., dog greeting)	0.098	0.018	5.429	0.000
Train the dog	0.046	0.018	2.560	0.010
Look after the dog	0.102	0.018	5.777	0.000
Dog playing	0.098	0.018	5.446	0.000
Talk to others because of the dog	0.066	0.018	3.699	0.000
Sensory problems (e.g., barking, housesoiling)	0.006	0.019	0.305	0.760
Dog out of control	-0.004	0.020	-0.192	0.847
Dog aggression	-0.021	0.026	-0.807	0.420
Other unwanted behaviours	-0.025	0.023	-1.083	0.279

Table S15. Statistics for the variable 'closeness to the dog' when assessing negative affect of high arousal

Activity	B	SE	Z value	Pr(>|Z|)
Simply having the dog	0.100	0.018	5.683	0.000
Maintenance (e.g., dog bills, vet visits)	0.058	0.018	3.218	0.001
Health problems	-0.039	0.021	-1.866	0.062

Dog presence	0.109	0.018	6.192	0.000
Exercise (e.g., dog walk)	0.100	0.018	5.630	0.000
Tactile interactions	0.111	0.019	5.958	0.000
Other close interactions (e.g., dog greeting)	0.086	0.018	4.824	0.000
Train the dog	0.048	0.018	2.681	0.007
Look after the dog	0.072	0.017	4.091	0.000
Dog playing	0.070	0.018	3.957	0.000
Talk to others because of the dog	0.065	0.018	3.667	0.000
Sensory problems (e.g., barking, housesoiling)	0.025	0.019	1.353	0.176
Dog out of control	-0.013	0.019	-0.672	0.502
Dog aggression	-0.024	0.026	-0.925	0.355
Other unwanted behaviours	-0.004	0.023	-0.190	0.850

Table S16. Statistics for the variable 'closeness to the dog' when assessing negative affect of low arousal

Activity	Estimate	Std error	Z value	Pr(>|Z|)
Simply having the dog	0.094	0.018	5.326	0.000
Maintenance (e.g., dog bills, vet visits)	0.044	0.019	2.346	0.019
Health problems	-0.024	0.021	-1.151	0.250
Dog presence	0.111	0.018	6.189	0.000
Exercise (e.g., dog walk)	0.092	0.018	5.150	0.000
Tactile interactions	0.119	0.019	6.337	0.000
Other close interactions (e.g., dog greeting)	0.095	0.018	5.293	0.000
Train the dog	0.082	0.018	4.508	0.000
Look after the dog	0.082	0.018	4.660	0.000
Dog playing	0.069	0.018	3.861	0.000
Talk to others because of the dog	0.071	0.018	4.009	0.000
Sensory problems (e.g., barking, housesoiling)	0.020	0.019	1.030	0.303
Dog out of control	-0.006	0.020	-0.291	0.771
Dog aggression	0.009	0.026	0.331	0.740
Other unwanted behaviours	-0.028	0.023	-1.246	0.213

Table S17. Statistics for the variable 'closeness to the dog' when assessing life satisfaction

Activity	Estimate	Std error	Z value	Pr(>|Z|)
Simply having the dog	0.103	0.019	5.429	0.000
Maintenance (e.g., dog bills, vet visits)	0.083	0.020	4.258	0.000
Health problems	-0.054	0.021	-2.617	0.009
Dog presence	0.099	0.019	5.315	0.000
Exercise (e.g., dog walk)	0.101	0.018	5.495	0.000
Tactile interactions	0.147	0.019	7.752	0.000
Other close interactions (e.g., dog greeting)	0.110	0.018	6.017	0.000
Train the dog	0.088	0.019	4.771	0.000
Look after the dog	0.099	0.018	5.466	0.000
Dog playing	0.081	0.018	4.434	0.000
Talk to others because of the dog	0.100	0.018	5.508	0.000
Sensory problems (e.g., barking, housesoiling)	0.006	0.021	0.305	0.761

Dog out of control	0.010	0.021	0.483	0.629
Dog aggression	0.003	0.028	0.122	0.903
Other unwanted behaviours	-0.029	0.025	-1.153	0.249

Table S18. Statistics for the variable 'closeness to the dog' when assessing autonomy

Activity	Estimate	Std error	Z value	Pr(>|Z|)
Simply having the dog	0.065	0.017	3.763	0.000
Maintenance (e.g., dog bills, vet visits)	0.069	0.019	3.728	0.000
Health problems	-0.011	0.021	-0.544	0.586
Dog presence	0.078	0.018	4.484	0.000
Exercise (e.g., dog walk)	0.084	0.018	4.744	0.000
Tactile interactions	0.094	0.019	5.039	0.000
Other close interactions (e.g., dog greeting)	0.085	0.018	4.625	0.000
Train the dog	0.058	0.018	3.186	0.001
Look after the dog	0.079	0.018	4.418	0.000
Dog playing	0.072	0.019	3.825	0.000
Talk to others because of the dog	0.070	0.018	3.909	0.000
Sensory problems (e.g., barking, housesoiling)	0.001	0.020	0.058	0.954
Dog out of control	-0.025	0.020	-1.271	0.204
Dog aggression	-0.028	0.026	-1.059	0.289
Other unwanted behaviours	-0.034	0.024	-1.454	0.146

Table S19. Statistics for the variable 'closeness to the dog' when assessing environmental mastery

Activity	Estimate	Std error	Z value	Pr(>|Z|)
Simply having the dog	0.069	0.017	3.996	0.000
Maintenance (e.g., dog bills, vet visits)	0.064	0.018	3.556	0.000
Health problems	-0.019	0.020	-0.966	0.334
Dog presence	0.073	0.017	4.168	0.000
Exercise (e.g., dog walk)	0.092	0.018	5.203	0.000
Tactile interactions	0.099	0.019	5.310	0.000
Other close interactions (e.g., dog greeting)	0.102	0.019	5.432	0.000
Train the dog	0.059	0.018	3.270	0.001
Look after the dog	0.069	0.018	3.922	0.000
Dog playing	0.052	0.019	2.825	0.005
Talk to others because of the dog	0.062	0.018	3.407	0.001
Sensory problems (e.g., barking, housesoiling)	-0.016	0.019	-0.829	0.407
Dog out of control	-0.032	0.020	-1.617	0.106
Dog aggression	-0.006	0.026	-0.233	0.815
Other unwanted behaviours	-0.026	0.023	-1.149	0.251

Table S20. Statistics for the variable 'closeness to the dog' when assessing personal growth

Activity	Estimate	Std error	Z value	Pr(>|Z|)
Simply having the dog	0.082	0.018	4.583	0.000
Maintenance (e.g., dog bills, vet visits)	0.073	0.020	3.636	0.000

Health problems	-0.026	0.021	-1.255	0.210
Dog presence	0.077	0.018	4.273	0.000
Exercise (e.g., dog walk)	0.086	0.018	4.729	0.000
Tactile interactions	0.096	0.019	4.951	0.000
Other close interactions (e.g., dog greeting)	0.104	0.020	5.275	0.000
Train the dog	0.069	0.018	3.728	0.000
Look after the dog	0.072	0.018	3.929	0.000
Dog playing	0.070	0.019	3.658	0.000
Talk to others because of the dog	0.092	0.019	4.960	0.000
Sensory problems (e.g., barking, housesoiling)	0.018	0.021	0.816	0.415
Dog out of control	-0.010	0.021	-0.476	0.634
Dog aggression	0.009	0.027	0.324	0.746
Other unwanted behaviours	-0.012	0.025	-0.483	0.629

Table S21. Statistics for the variable 'closeness to the dog' when assessing positive relations

Activity	Estimate	Std error	Z value	Pr(>|Z|)
Simply having the dog	0.068	0.018	3.872	0.000
Maintenance (e.g., dog bills, vet visits)	0.063	0.020	3.144	0.002
Health problems	-0.006	0.021	-0.284	0.777
Dog presence	0.067	0.018	3.774	0.000
Exercise (e.g., dog walk)	0.084	0.018	4.716	0.000
Tactile interactions	0.093	0.019	4.870	0.000
Other close interactions (e.g., dog greeting)	0.099	0.019	5.078	0.000
Train the dog	0.058	0.018	3.162	0.002
Look after the dog	0.064	0.019	3.354	0.001
Dog playing	0.056	0.018	3.098	0.002
Talk to others because of the dog	0.084	0.018	4.637	0.000
Sensory problems (e.g., barking, housesoiling)	0.011	0.020	0.532	0.595
Dog out of control	0.005	0.020	0.268	0.789
Dog aggression	-0.022	0.026	-0.830	0.407
Other unwanted behaviours	0.009	0.024	0.364	0.716

Table S22. Statistics for the variable 'closeness to the dog' when assessing purpose in life

Activity	Estimate	Std error	Z value	Pr(>|Z|)
Simply having the dog	0.091	0.018	5.026	0.000
Maintenance (e.g., dog bills, vet visits)	0.093	0.020	4.637	0.000
Health problems	0.006	0.022	0.275	0.784
Dog presence	0.088	0.018	4.852	0.000
Exercise (e.g., dog walk)	0.109	0.018	5.976	0.000
Tactile interactions	0.088	0.019	4.635	0.000
Other close interactions (e.g., dog greeting)	0.099	0.019	5.133	0.000
Train the dog	0.066	0.019	3.538	0.000
Look after the dog	0.065	0.018	3.553	0.000
Dog playing	0.062	0.019	3.265	0.001
Talk to others because of the dog	0.095	0.019	5.027	0.000

Sensory problems (e.g., barking, housesoiling)	0.032	0.023	1.394	0.163
Dog out of control	0.017	0.022	0.757	0.449
Dog aggression	0.018	0.029	0.620	0.535
Other unwanted behaviours	0.017	0.026	0.636	0.525

Table S23. Statistics for the variable 'closeness to the dog' when assessing self-acceptance

**Activity**	**Estimate**	**Std error**	**Z value**	**Pr(>|Z|)**
Simply having the dog	0.092	0.018	5.110	0.000
Maintenance (e.g., dog bills, vet visits)	0.089	0.021	4.267	0.000
Health problems	-0.040	0.021	-1.882	0.060
Dog presence	0.093	0.018	5.123	0.000
Exercise (e.g., dog walk)	0.109	0.018	5.989	0.000
Tactile interactions	0.095	0.019	5.127	0.000
Other close interactions (e.g., dog greeting)	0.097	0.019	5.197	0.000
Train the dog	0.072	0.019	3.863	0.000
Look after the dog	0.077	0.019	4.074	0.000
Dog playing	0.083	0.019	4.456	0.000
Talk to others because of the dog	0.073	0.018	3.974	0.000
Sensory problems (e.g., barking, housesoiling)	-0.007	0.021	-0.313	0.754
Dog out of control	-0.017	0.021	-0.826	0.409
Dog aggression	0.003	0.027	0.121	0.904
Other unwanted behaviours	-0.010	0.025	-0.393	0.694

Confidence intervals at lower and higher 'closeness to the dog'

Note: each line of activity contains the confidence intervals for lower and higher 'closeness to the dog'

Table S24. 99% Confidence intervals for positive affect of high arousal at lower and higher 'closeness to the dog'

Activity	CI of lower closeness to the dog			CI of higher closeness to the dog		
	Lower	Upper	Est.mean	Lower	Upper	Est.mean
Simply having the dog	2.086	2.381	2.234	2.551	2.689	2.620
Maintenance (e.g., dog bills, vet visits)	-0.088	0.205	0.058	0.162	0.359	0.260
Health problems	-2.074	-1.710	-1.892	-2.294	-2.086	-2.190
Dog presence	2.046	2.336	2.191	2.503	2.645	2.574
Exercise (e.g., dog walk)	1.725	2.062	1.893	2.236	2.416	2.326
Tactile interactions	1.923	2.232	2.077	2.424	2.582	2.503
Other close interactions (e.g., dog greeting)	1.796	2.103	1.949	2.245	2.415	2.330
Train the dog	1.478	1.826	1.652	1.868	2.078	1.973
Look after the dog	0.812	1.142	0.977	1.396	1.617	1.507
Dog playing	2.092	2.353	2.222	2.492	2.629	2.560
Talk to others because of the dog	1.455	1.778	1.617	1.789	1.988	1.888
Sensory problems (e.g., barking, housesoiling)	-0.722	-0.454	-0.588	-0.636	-0.457	-0.547
Dog out of control	-1.130	-0.836	-0.983	-1.086	-0.890	-0.988
Dog aggression	-1.627	-1.176	-1.402	-1.563	-1.260	-1.412
Other unwanted behaviours	-0.832	-0.526	-0.679	-0.930	-0.719	-0.825

Table S25. 99% Confidence intervals for positive affect of low arousal at lower and higher 'closeness to the dog'

Activity	CI of lower closeness to the dog			CI of higher closeness to the dog		
	Lower	Upper	Est.mean	Lower	Upper	Est.mean
Simply having the dog	1.358	1.756	1.557	1.996	2.213	2.104
Maintenance (e.g., dog bills, vet visits)	-0.187	0.089	-0.049	-0.010	0.173	0.081
Health problems	-2.140	-1.778	-1.959	-2.164	-1.941	-2.053
Dog presence	1.638	2.010	1.824	2.213	2.407	2.310
Exercise (e.g., dog walk)	1.430	1.815	1.622	1.974	2.192	2.083
Tactile interactions	2.127	2.412	2.269	2.528	2.670	2.599
Other close interactions (e.g., dog greeting)	1.351	1.705	1.528	1.843	2.054	1.948
Train the dog	0.813	1.213	1.013	1.115	1.378	1.246
Look after the dog	0.695	1.043	0.869	1.217	1.446	1.332
Dog playing	1.234	1.592	1.413	1.735	1.952	1.844
Talk to others because of the dog	0.787	1.147	0.967	1.153	1.390	1.271
Sensory problems (e.g., barking, housesoiling)	-1.135	-0.868	-1.001	-1.072	-0.894	-0.983
Dog out of control	-1.445	-1.145	-1.295	-1.408	-1.207	-1.308
Dog aggression	-1.835	-1.390	-1.612	-1.844	-1.547	-1.696
Other unwanted behaviours	-1.079	-0.766	-0.922	-1.107	-0.893	-1.000

Table S26. 99% Confidence intervals for negative affect of high arousal at lower and higher 'closeness to the dog

Activity	CI of lower closeness to the dog			CI of higher closeness to the dog		
	Lower	Upper	Est.mean	Lower	Upper	Est.mean
Simply having the dog	0.440	0.920	0.680	1.140	1.440	1.290
Maintenance (e.g., dog bills, vet visits)	-0.602	-0.308	-0.455	-0.336	-0.138	-0.237
Health problems	-1.957	-1.458	-1.707	-2.056	-1.759	-1.907

Dog presence	0.599	1.079	0.839	1.347	1.635	1.491
Exercise (e.g., dog walk)	0.607	1.082	0.844	1.292	1.584	1.438
Tactile interactions	1.191	1.669	1.430	1.894	2.154	2.024
Other close interactions (e.g., dog greeting)	0.830	1.264	1.047	1.376	1.642	1.509
Train the dog	0.257	0.692	0.475	0.597	0.889	0.743
Look after the dog	0.140	0.502	0.321	0.541	0.787	0.664
Dog playing	0.907	1.342	1.124	1.370	1.638	1.504
Talk to others because of the dog	0.408	0.796	0.602	0.799	1.053	0.926
Sensory problems (e.g., barking, housesoiling)	-1.143	-0.847	-0.995	-1.003	-0.802	-0.902
Dog out of control	-1.508	-1.165	-1.337	-1.503	-1.276	-1.390
Dog aggression	-1.771	-1.253	-1.512	-1.791	-1.452	-1.621
Other unwanted behaviours	-1.216	-0.859	-1.037	-1.172	-0.933	-1.053

Table S27. 99% Confidence intervals for negative affect of low arousal at lower and higher 'closeness to the dog

Activity	CI of lower closeness to the dog			CI of higher closeness to the dog		
	Lower	Upper	Est.mean	Lower	Upper	Est.mean
Simply having the dog	0.776	1.247	1.012	1.419	1.703	1.561
Maintenance (e.g., dog bills, vet visits)	-0.217	0.045	-0.086	-0.034	0.141	0.053
Health problems	-2.017	-1.520	-1.769	-2.045	-1.741	-1.893
Dog presence	0.844	1.308	1.076	1.559	1.829	1.694
Exercise (e.g., dog walk)	0.856	1.297	1.077	1.443	1.712	1.577
Tactile interactions	1.191	1.658	1.425	1.914	2.167	2.041
Other close interactions (e.g., dog greeting)	0.977	1.398	1.187	1.549	1.803	1.676
Train the dog	0.386	0.797	0.591	0.880	1.154	1.017
Look after the dog	0.220	0.572	0.396	0.656	0.896	0.776
Dog playing	0.975	1.407	1.191	1.425	1.690	1.558
Talk to others because of the dog	0.520	0.905	0.713	0.937	1.189	1.063
Sensory problems (e.g., barking, housesoiling)	-0.553	-0.307	-0.430	-0.453	-0.291	-0.372
Dog out of control	-0.791	-0.485	-0.638	-0.762	-0.555	-0.658
Dog aggression	-1.327	-0.818	-1.072	-1.205	-0.862	-1.033
Other unwanted behaviours	-0.758	-0.398	-0.578	-0.802	-0.559	-0.680

Table S28. 99% Confidence intervals for life satisfaction at lower and higher 'closeness to the dog'

Activity	CI of lower closeness to the dog			CI of higher closeness to the dog		
	Lower	Upper	Est.mean	Lower	Upper	Est.mean
Simply having the dog	1.760	2.098	1.929	2.220	2.405	2.313
Maintenance (e.g., dog bills, vet visits)	0.091	0.342	0.216	0.379	0.563	0.471
Health problems	-1.110	-0.746	-0.928	-1.267	-1.026	-1.147
Dog presence	1.693	2.028	1.860	2.143	2.331	2.237
Exercise (e.g., dog walk)	1.448	1.802	1.625	1.944	2.154	2.049
Tactile interactions	1.479	1.825	1.652	2.136	2.333	2.235
Other close interactions (e.g., dog greeting)	1.315	1.662	1.488	1.847	2.061	1.954
Train the dog	1.158	1.526	1.342	1.621	1.855	1.738
Look after the dog	0.840	1.173	1.006	1.320	1.549	1.434
Dog playing	1.384	1.727	1.555	1.789	2.005	1.897
Talk to others because of the dog	1.100	1.439	1.269	1.584	1.801	1.693
Sensory problems (e.g., barking, housesoiling)	-0.284	-0.072	-0.178	-0.235	-0.092	-0.163
Dog out of control	-0.491	-0.245	-0.368	-0.423	-0.258	-0.341
Dog aggression	-0.787	-0.391	-0.589	-0.712	-0.444	-0.578

Other unwanted behaviours	-0.406	-0.148	-0.277	-0.439	-0.253	-0.346

Table S29. 99% Confidence intervals for autonomy at lower and higher 'closeness to the dog'

Activity	CI of lower closeness to the dog			CI of higher closeness to the dog		
	Lower	Upper	Est.mean	Lower	Upper	Est.mean
Simply having the dog	0.315	0.743	0.529	0.755	1.039	0.897
Maintenance (e.g., dog bills, vet visits)	-0.076	0.202	0.063	0.207	0.404	0.305
Health problems	-0.732	-0.394	-0.563	-0.713	-0.495	-0.604
Dog presence	0.379	0.781	0.580	0.860	1.130	0.995
Exercise (e.g., dog walk)	0.558	0.947	0.752	1.047	1.309	1.178
Tactile interactions	0.468	0.768	0.618	0.874	1.104	0.989
Other close interactions (e.g., dog greeting)	0.373	0.690	0.532	0.766	0.998	0.882
Train the dog	0.537	0.905	0.721	0.867	1.116	0.992
Look after the dog	0.129	0.467	0.298	0.530	0.767	0.649
Dog playing	0.424	0.722	0.573	0.735	0.957	0.846
Talk to others because of the dog	0.290	0.621	0.456	0.642	0.868	0.755
Sensory problems (e.g., barking, housesoiling)	-0.546	-0.300	-0.423	-0.503	-0.337	-0.420
Dog out of control	-0.743	-0.468	-0.606	-0.783	-0.592	-0.687
Dog aggression	-1.010	-0.588	-0.799	-1.051	-0.758	-0.904
Other unwanted behaviours	-0.624	-0.307	-0.466	-0.685	-0.461	-0.573

Table S30. 99% Confidence intervals for environmental mastery at lower and higher 'closeness to the dog'

Activity	CI of lower closeness to the dog			CI of higher closeness to the dog		
	Lower	Upper	Est.mean	Lower	Upper	Est.mean
Simply having the dog	0.157	0.546	0.351	0.579	0.843	0.711
Maintenance (e.g., dog bills, vet visits)	-0.176	0.137	-0.019	0.131	0.344	0.237
Health problems	-1.542	-1.164	-1.353	-1.554	-1.315	-1.435
Dog presence	0.249	0.619	0.434	0.665	0.920	0.793
Exercise (e.g., dog walk)	0.360	0.730	0.545	0.866	1.117	0.991
Tactile interactions	0.431	0.718	0.575	0.839	1.061	0.950
Other close interactions (e.g., dog greeting)	0.271	0.556	0.414	0.685	0.906	0.796
Train the dog	0.850	1.223	1.037	1.192	1.435	1.314
Look after the dog	0.295	0.633	0.464	0.656	0.888	0.772
Dog playing	0.405	0.698	0.551	0.641	0.850	0.745
Talk to others because of the dog	0.281	0.588	0.435	0.571	0.784	0.678
Sensory problems (e.g., barking, housesoiling)	-0.781	-0.521	-0.651	-0.789	-0.613	-0.701
Dog out of control	-1.101	-0.798	-0.950	-1.167	-0.961	-1.064
Dog aggression	-1.404	-0.946	-1.175	-1.354	-1.045	-1.200
Other unwanted behaviours	-0.871	-0.536	-0.703	-0.905	-0.678	-0.792

Table S31. 99% Confidence intervals for personal growth at lower and higher 'closeness to the dog'

Activity	CI of lower closeness to the dog			CI of higher closeness to the dog		
	Lower	Upper	Est.mean	Lower	Upper	Est.mean
Simply having the dog	0.998	1.353	1.175	1.430	1.661	1.546
Maintenance (e.g., dog bills, vet visits)	0.124	0.353	0.239	0.352	0.526	0.439
Health problems	-0.399	-0.071	-0.235	-0.436	-0.221	-0.328
Dog presence	0.741	1.075	0.908	1.125	1.355	1.240

Exercise (e.g., dog walk)	0.749	1.083	0.916	1.171	1.405	1.288
Tactile interactions	0.474	0.759	0.617	0.858	1.084	0.971
Other close interactions (e.g., dog greeting)	0.386	0.647	0.517	0.763	0.980	0.872
Train the dog	1.025	1.387	1.206	1.396	1.633	1.514
Look after the dog	0.538	0.837	0.688	0.860	1.077	0.968
Dog playing	0.512	0.801	0.657	0.805	1.021	0.913
Talk to others because of the dog	0.568	0.870	0.719	0.969	1.189	1.079
Sensory problems (e.g., barking, housesoiling)	-0.142	0.065	-0.038	-0.069	0.069	0.000
Dog out of control	-0.226	0.024	-0.101	-0.212	-0.044	-0.128
Dog aggression	-0.386	0.011	-0.187	-0.292	-0.023	-0.158
Other unwanted behaviours	-0.186	0.102	-0.042	-0.170	0.024	-0.073

Table S32. 99% Confidence intervals for positive relations with others at lower and higher 'closeness to the dog'

Activity	CI of lower closeness to the dog			CI of higher closeness to the dog		
	Lower	Upper	Est.mean	Lower	Upper	Est.mean
Simply having the dog	1.081	1.439	1.260	1.457	1.686	1.571
Maintenance (e.g., dog bills, vet visits)	0.161	0.386	0.274	0.359	0.527	0.443
Health problems	-0.282	0.030	-0.126	-0.245	-0.046	-0.146
Dog presence	0.954	1.309	1.131	1.320	1.556	1.438
Exercise (e.g., dog walk)	0.789	1.144	0.966	1.232	1.468	1.350
Tactile interactions	0.486	0.781	0.633	0.876	1.104	0.990
Other close interactions (e.g., dog greeting)	0.411	0.679	0.545	0.782	0.997	0.890
Train the dog	0.807	1.159	0.983	1.121	1.358	1.240
Look after the dog	0.379	0.663	0.521	0.646	0.855	0.750
Dog playing	0.808	1.131	0.970	1.091	1.313	1.202
Talk to others because of the dog	1.301	1.657	1.479	1.733	1.951	1.842
Sensory problems (e.g., barking, housesoiling)	-0.446	-0.209	-0.328	-0.378	-0.220	-0.299
Dog out of control	-0.656	-0.374	-0.515	-0.592	-0.404	-0.498
Dog aggression	-0.962	-0.523	-0.742	-0.977	-0.678	-0.827
Other unwanted behaviours	-0.448	-0.161	-0.304	-0.376	-0.186	-0.281

Table S33. 99% Confidence intervals for purpose in life at lower and higher 'closeness to the dog'

Activity	CI of lower closeness to the dog			CI of higher closeness to the dog		
	Lower	Upper	Est.mean	Lower	Upper	Est.mean
Simply having the dog	1.144	1.491	1.318	1.602	1.828	1.715
Maintenance (e.g., dog bills, vet visits)	0.178	0.422	0.300	0.484	0.677	0.581
Health problems	-0.346	-0.017	-0.181	-0.265	-0.058	-0.161
Dog presence	0.946	1.289	1.117	1.387	1.621	1.504
Exercise (e.g., dog walk)	0.784	1.111	0.947	1.297	1.530	1.414
Tactile interactions	0.591	0.896	0.744	0.975	1.206	1.090
Other close interactions (e.g., dog greeting)	0.536	0.823	0.680	0.938	1.164	1.051
Train the dog	0.960	1.311	1.135	1.304	1.543	1.423
Look after the dog	0.765	1.088	0.927	1.083	1.310	1.196
Dog playing	0.618	0.916	0.767	0.890	1.111	1.000
Talk to others because of the dog	0.546	0.846	0.696	0.952	1.178	1.065
Sensory problems (e.g., barking, housesoiling)	-0.063	0.122	0.029	0.025	0.150	0.088
Dog out of control	-0.146	0.097	-0.025	-0.064	0.098	0.017

Dog aggression	-0.161	0.180	0.009	-0.058	0.174	0.058
Other unwanted behaviours	-0.099	0.174	0.038	-0.016	0.169	0.077

Table S33. 99% Confidence intervals for self-acceptance at lower and higher 'closeness to the dog'

Activity	CI of lower closeness to the dog			CI of higher closeness to the dog		
	Lower	Upper	Est.mean	Lower	Upper	Est.mean
Simply having the dog	0.896	1.252	1.074	1.374	1.608	1.491
Maintenance (e.g., dog bills, vet visits)	0.129	0.353	0.241	0.390	0.570	0.480
Health problems	-0.302	0.024	-0.139	-0.384	-0.171	-0.278
Dog presence	0.875	1.219	1.047	1.340	1.572	1.456
Exercise (e.g., dog walk)	0.689	1.021	0.855	1.210	1.442	1.326
Tactile interactions	0.880	1.208	1.044	1.330	1.564	1.447
Other close interactions (e.g., dog greeting)	0.727	1.045	0.886	1.170	1.404	1.287
Train the dog	0.631	0.981	0.806	1.000	1.242	1.121
Look after the dog	0.523	0.813	0.668	0.847	1.065	0.956
Dog playing	0.638	0.944	0.791	1.008	1.236	1.122
Talk to others because of the dog	0.608	0.925	0.767	0.952	1.175	1.064
Sensory problems (e.g., barking, housesoiling)	-0.137	0.085	-0.026	-0.117	0.033	-0.042
Dog out of control	-0.269	0.003	-0.133	-0.277	-0.092	-0.184
Dog aggression	-0.444	-0.035	-0.240	-0.367	-0.090	-0.228
Other unwanted behaviours	-0.214	0.064	-0.075	-0.195	-0.005	-0.100
